# Sociodemographic and behavioral influences on multimorbidity among adult residents of northeastern China

**DOI:** 10.1186/s12889-022-12722-y

**Published:** 2022-02-18

**Authors:** Jikang Shi, Yanbo Guo, Zhen Li, Zhuoshuai Liang, Lingfeng Pan, Yang Yu, Wenfei Zhu, Aiyu Shao, Wenjun Chen, Chao Gao, Siyu Liu, Yawen Liu, Yi Cheng

**Affiliations:** 1 Department of Epidemiology and Biostatistics, School of Public Health of Jilin University, Changchun, 130021 China; 2 The Cardiovascular Center, The First Hospital of Jilin University, Changchun, 130021 Jilin China

**Keywords:** Adults, China, Influencing factors, Multimorbidity

## Abstract

**Background:**

Multimorbidity is defined as two or more chronic health conditions existing in an individual simultaneously. Multimorbidity has been associated with poor conditions, such as higher health care costs and the poor quality of life. Thus, identifying the risk factors of the multimorbidity is required for multimorbidity prevention.

**Methods:**

This study was based on the Comprehensive Demonstration Research Project of Major Chronic Noncommunicable Disease Prevention and Control Technology in Northeast China initiated by China Medical University. The investigation was a cross-sectional study under a multistage stratified cluster random sampling design. Associations between multimorbidity and sociodemographic and behavioral factors in adult residents were investigated using univariate analysis and multivariate logistic regression analysis.

**Results:**

A total of 6706 participants were enrolled in this investigation, and the prevalence of multimorbidity was 21.2% among the adult residents of northeastern China. There existed differences of association between age and multimorbidity risks (65–69 years old: OR = 3.53, 95%CI: 2.04–6.12; 70–74 years old: OR = 5.26, 95%CI: 3.02–9.17). Participants who are overweight had significantly high multimorbidity risk (OR = 2.76, 95%CI: 1.50–5.24). Family history of hypertension and family history of diabetes were significantly associated with high multimorbidity risk (family history of hypertension: OR = 2.34, 95%CI: 1.96–2.79; family history of diabetes: OR = 1.77, 95%CI: 1.38–2.26). Compared with the frequency of fatigue (< 1 time/week or 1–2 times/week), that (≥3 times/week) was associated with high multimorbidity risk (OR = 1.39, 95%CI: 1.07–1.81). For fresh fruit consumption, compared with eating fruits regularly, eating rarely had a higher risk of multimorbidity (OR = 2.33, 95%CI: 1.90–2.85).

**Conclusions:**

Sociodemographic indices (age, BMI, family history of hypertension, and family history of diabetes) and behavioral indices (fatigue status and fresh fruit consumption) increase the risks of multimorbidity. This study provides a necessary route to prevent and control multimorbidity in northeast China.

**Supplementary Information:**

The online version contains supplementary material available at 10.1186/s12889-022-12722-y.

## Introduction

Multimorbidity is defined as two or more chronic health conditions existing in an individual simultaneously [1–4]. Multimorbidity increases with aging [5]. Aging is a risk factor of multimorbidity; moreover, the number and proportion of the elderly are increasing sharply in China. Thus, China has to face a heavy burden of the multimorbidity in future decades [6, 7].

Multimorbidity has been associated with adverse events, including longer hospitalizations, multiple medical treatments, more complications, psychological distress, higher health care costs, and the poorer quality of life [8–15]. A higher number of chronic conditions in an individual is associated with higher mortality [16–18]. In addition, multimorbidity is associated with a higher risk of unemployment [19], and multimorbidity leads to a substantial economic burden on health care systems [20–22]. Therefore, identifying the risk factors for multimorbidity to further address the major public health problems.

To date, the prevalence and pattern of multimorbidity has been investigated worldwide. The prevalence of multimorbidity are reported as following: 28% in Americans [23], 37.1% in Australia [24], 58.2% in women who are more than 50 years old in Brazil [25], and 6.4–76.5% in the population aged 60 years or more in China [26]. The difference of multimorbidity prevalence may arise from population, data sources, and eating habits from different areas. The major patterns of multimorbidity are identified as cardiovascular and metabolic diseases, mental health problems, and musculoskeletal disorders in the elderly who lived in Europe, the United States (U.S.), and Australia [27]. In contrast, cardiopulmonary-mental-degenerative disorder and cerebrovascular-metabolic disorder are the patterns identified in China [28]. Indeed, different methods, population, and chronic diseases have been used in defining multimorbidity pattern, affording that there exists no consensus on the determination and classification of multimorbidity pattern.

The prevention and control of chronic disease are necessary for multimorbidity management, underscoring the identification of risk factors of the multimorbidity. The risk factors for multimorbidity have been identified in studies, including age, female, and low socioeconomic status [29–31]. Moreover, influencing factors of multimorbidity, such as racial and ethnic, remain controversial [32, 33]. Thus, more studies are needed to investigate risk factors for multimorbidity. In this paper, we investigated the prevalence of multimorbidity and further evaluated the sociodemographic and behavioral influences on multimorbidity among adult residents to identify the risk factors for multimorbidity in Changchun, China.

## Materials and methods

### Ethical statement

The study was approved by the Ethics Committee of China Medical University. The study protocol was performed in accordance with the principles outlined in the Declaration of Helsinki, and informed consent was collected from each of participants.

### Study population

The study was affiliated to the Comprehensive Demonstration Research Project of Major Chronic Noncommunicable Disease Prevention and Control Technology in Northeast China initiated by China Medical University. The investigation, which was conducted from January 1, 2019 to November 31, 2019, was a cross-sectional study under a multistage stratified cluster random sampling design. The data were collected from residents of 10 districts in Changchun city, Jilin Province. The adult residents were enrolled according to following inclusion criteria: (1) over the age of 35 years; (2) with registered permanent residence (a record officially identifying area residents); (3) living in Changchun for more than 6 months; (4) with consciousness and no communication barriers; (5) good compliance. The exclusion criteria satisfied the followings: (1) incomplete information; (2) data with outliers. (Supplemental Fig. 1).

### Questionnaire and health examination

The questionnaire was designed by the China Medical University and the School of Public Health, Jilin University. Direct face-to-face interview survey was performed by uniformly trained investigators. Questionnaires and data of anthropometric measurements were collected from each participant. Demographic information (sex, age, ethnicity, marital status, occupation, annual income, and level of education), health behaviors (smoking, drinking, diet, sleep status, and physical activity), and history of chronic diseases (hypertension, diabetes, coronary heart disease, and stroke), were collected from the questionnaires. In addition, the information of anthropometric measurements (height, weight, blood pressure, fasting blood glucose, and blood lipids) were obtained from health examination. Every physical measurement was checked by two medical staffs together. Blood samples were collected and transported to a central laboratory via a cold chain transport system.

### Statistical analysis

Constituent ratio was used to represent the composition of prevalence of chronic diseases for classified participants according to sociodemographic and behavioral characteristics. *Chi-square* (*χ*^2^) test was used to identify the relationship of multimorbidity with sociodemographic and behavioral characteristics. Multivariate logistic regression was performed to analyze odds ratios (OR) for multimorbidity. The predictive models were built on the basis of risk factors and visualized using nomograms, and the performance of our models was evaluated using the Harrell’s concordance index (c-index). SPSS version 24.0 and R version 4.1.0 were used for statistical analysis, and *P*-values < 0.05 was considered statistically significant.

## Results

A total of 6706 participants were enrolled in this investigation. The mean age of the participants was 68.79 years old, and the prevalence of multimorbidity was 21.2%. The participants were divided into four groups according to the number of chronic disease (1 disease, 2 diseases, and ≥ 3 diseases), and corresponding data of prevalence are showed in Table 1. Significant differences of prevalence classified by number of chronic diseases existed in age, BMI, marital status, family history of hypertension, family history of diabetes, educational level, occupation, annual income, physical exercise, sleep status, fatigue status, stay up late, salt taste, edible oil taste, carbonated drinks, fresh fruit consumption, meat consumption (red meat and poultry), consumption of fish, and consumption of eggs and beans (*P* < 0.05).

**Table 1:** Prevalence of number of chronic diseases by sociodemographic and behavioral characteristics of the study population

Variables	Total (n)	0disease		1disaese		2diseases		≥3diseases		χ^2^	*P*
		n	%	n	%	n	%	n	%		
Sex											
Male	2677	1082	40.4	1045	39.0	450	16.8	100	3.7	3.481	0.323
Female	4029	1551	38.5	1604	39.8	699	17.3	175	4.3		
Age (year)											
≤ 64	191	114	59.7	62	32.5	12	6.3	3	1.6	94.721	<0.001*
65–69	3929	1643	41.8	1519	38.7	637	16.2	130	3.3		
70–74	2586	876	33.9	1068	41.3	500	19.3	142	5.5		
Ethnicity											
Han	6517	2558	39.3	2576	39.5	1121	17.2	262	4.0	4.316	0.229
Non-Han	189	75	39.7	73	38.6	28	14.8	13	6.9		
BMI											
Underweight	102	55	53.9	36	35.3	9	8.8	2	2.0	163.603	<0.001*
Normal	2568	1230	47.9	911	35.5	351	13.7	76	3.0		
Overweight	3961	1316	33.2	1674	42.3	775	19.6	196	4.9		
Obese	75	32	42.7	28	37.3	14	18.7	1	1.3		
Marital status											
Married/Cohabitation	35	18	51.4	14	40.0	3	8.6	0	0.0	14.601	0.024*
Unmarried	5923	2357	39.8	2325	39.3	1007	17	234	4.0		
Divorced/ Separated	748	258	34.5	310	41.4	139	18.6	41	5.5		
Family history of hypertension											
No	5940	2450	41.2	2353	39.6	949	16	188	3.2	203.931	<0.001*
Yes	766	183	23.9	296	38.6	200	26.1	87	11.4		
Family history of diabetes											
No	6064	2427	40	2418	39.9	1004	16.6	215	3.5	109.425	<0.001*
Yes	349	83	23.8	133	38.1	89	25.5	44	12.6		
Educational level											
Primary school or below	162	52	32.1	69	42.6	36	22.2	5	3.1	20.846	0.013*
Junior middle school	861	300	34.8	378	43.9	151	17.5	32	3.7		
Senior middle schoo	2280	879	38.6	915	40.1	388	17	98	4.3		
Undergraduate or above	3403	1402	41.2	1287	37.8	574	16.9	140	4.1		
Occupation											
Agriculture	321	102	31.8	140	43.6	62	19.3	17	5.3	61.841	<0.001*
Industry	512	212	41.4	198	38.7	79	15.4	23	4.5		
Individual business and service industry	521	231	44.3	192	36.9	86	16.5	12	2.3		
Agency and business unit	592	291	49.2	192	32.4	88	14.9	21	3.5		
Retirement	3638	1410	38.8	1432	39.4	633	17.4	163	4.5		
Unemployment	664	231	34.8	302	45.5	108	16.3	23	3.5		
Other	458	156	34.1	193	42.1	93	20.3	16	3.5		
Annual income (¥)											
< 10,000	563	196	34.8	240	42.6	91	16.2	36	6.4	89.176	<0.001*
10,000 ~ 30,000	2817	960	34.1	1237	43.9	508	18	112	4.0		
30,000 ~ 50,000	2765	1213	43.9	987	35.7	464	16.8	101	3.7		
≥ ~ 50,000	561	264	47.1	185	33.0	86	15.3	26	4.6		
Physical exercise											
Every day	4912	1893	38.5	2002	40.8	827	16.8	190	3.9	30.286	0.003*
3-4 days/week	220	109	49.5	69	31.4	35	15.9	7	3.2		
2-3 days/week	200	96	48	62	31.0	30	15	12	6.0		
1-2 days/month	39	16	41	15	38.5	5	12.8	3	7.7		
Never	1335	519	38.9	501	37.5	252	18.9	63	4.7		
Sleep status											
Worse	76	20	26.3	34	44.7	16	21.1	6	7.9	28.86	0.004*
Poor	622	216	34.7	237	38.1	131	21.1	38	6.1		
Average	2015	796	39.5	787	39.1	343	17	89	4.4		
Good	3642	1453	39.9	1457	40.0	600	16.5	132	3.6		
Excellent	351	148	42.2	134	38.2	59	16.8	10	2.8		
Fatigueness status (time/week)											
<1	5304	2096	39.5	2114	39.9	887	16.7	207	3.9	22.967	0.001*
1–2	1061	418	39.4	420	39.6	178	16.8	45	4.2		
≥ 3	341	119	34.9	115	33.7	84	24.6	23	6.7		
Stay up late											
Often	214	96	44.9	70	32.7	40	18.7	8	3.7	44.007	<0.001*
Sometimes	479	229	47.8	162	33.8	71	14.8	17	3.5		
Rarely	1243	548	44.1	447	36.0	194	15.6	54	4.3		
Never	4770	1760	36.9	1970	41.3	844	17.7	196	4.1		
Smoking status											
Smoker	945	366	38.7	377	39.9	161	17	41	4.3	0.28	0.964
Non-smoker	5761	2267	39.4	2272	39.4	988	17.1	234	4.1		
Status of alcohol drinking											
Drinker	815	325	39.9	329	40.4	132	16.2	29	3.6	1.407	0.704
Non-drinker	5891	2308	39.2	2320	39.4	1017	17.3	246	4.2		
Salt taste											
Salty	427	181	42.4	134	31.4	85	19.9	27	6.3	96.487	<0.001*
Insipid	1411	480	34	548	38.8	275	19.5	108	7.7		
Appropriate	4868	1972	40.5	1967	40.4	789	16.2	140	2.9		
Edible oil taste											
Greasy	278	120	43.2	89	32.0	56	20.1	13	4.7	77.108	<0.001*
Thin	1330	488	36.7	486	36.5	251	18.9	105	7.9		
Appropriate	5098	2025	39.7	2074	40.7	842	16.5	157	3.1		
Carbonated drinks											
Yes	111	60	54.1	39	35.1	10	9	2	1.8	12.637	0.005*
No	6595	2573	39	2610	39.6	1139	17.3	273	4.1		
Fresh fruit consumption											
Often/Always	4777	2002	41.9	1850	38.7	732	15.3	193	4.0	123.52	<0.001*
Sometimes	1352	505	37.4	550	40.7	245	18.1	52	3.8		
Rarely/Never	577	126	21.8	249	43.2	172	29.8	30	5.2		
Meat consumption (red meet)											
Often/Always	1829	741	40.5	729	39.9	287	15.7	72	3.9	14.157	0.028*
Sometimes	3593	1441	40.1	1384	38.5	621	17.3	147	4.1		
Rarely/Never	1284	451	35.1	536	41.7	241	18.8	56	4.4		
Meat consumption (poultry)											
Often/Always	1375	570	41.5	548	39.9	209	15.2	48	3.5	24.06	0.001*
Sometimes	3810	1535	40.4	1467	38.6	644	16.9	155	4.1		
Rarely/Never	1530	528	34.5	634	41.4	296	19.3	72	4.7		
Consumption of fish											
Often/Always	747	322	43.1	281	37.6	113	15.1	31	4.1	23.499	0.001*
Sometimes	3881	1580	40.7	1500	38.6	648	16.7	153	3.9		
Rarely/Never	2078	731	35.2	868	41.8	388	18.7	91	4.4		
Consumption of eggs and beans											
Often/Always	3837	1569	40.9	1499	39.1	609	15.9	160	4.2	26.452	<0.001*
Sometimes	2298	853	37.1	927	40.3	440	19.1	78	3.4		
Rarely/Never	571	211	37	223	39.1	100	17.5	37	6.5		
Consumption of milk											
Often/Always	2974	1205	40.5	1148	38.6	483	16.2	138	4.6	12.477	0.052
Sometimes	2265	888	39.2	891	39.3	407	18	79	3.5		
Rarely/Never	1467	540	36.8	610	41.6	259	17.7	58	4.0		
Consumption of rice											
Often/Always	5394	2144	39.7	2126	39.4	906	16.8	218	4.0	10.382	0.109
Sometimes	1060	411	38.8	417	39.3	186	17.5	46	4.3		
Rarely/Never	252	78	31	106	42.1	57	22.6	11	4.4		

*P < 0.05

We used univariate analysis to investigate the influencing factors of multimorbidity on the basis of 26 independent variables listed in the questionnaire, finding that multimorbidity was associated with age, BMI, marital status, family history of hypertension, family history of diabetes, sleep status, fatigue status, salt taste, edible oil taste, carbonated drinks, fresh fruit consumption, meat consumption (poultry), consumption of fish, and consumption of eggs and beans (*P* < 0.05) (Table 2). The prevalence of multimorbidity increased with aging (*P* < 0.001). The prevalence of multimorbidity in participants with underweight, normal weight, overweight, or obese was 10.8, 30.0, 24.5, and 20.0%, correspondingly (*P* < 0.001). There were the significant differences of prevalence in married/cohabitation, unmarried, and divorced/separated (8.6, 21.0, and 24.1%, respectively) (*P* = 0.027). The prevalence of multimorbidity in participants with family history of hypertension/diabetes was significantly higher than that in participants without the respective/corresponding one (*P* < 0.001). The prevalence of multimorbidity increased with the deteriorating of sleep status (*P* < 0.001). The prevalence of multimorbidity increased with the increasing frequency of fatigue (*P* < 0.001). For salt consumption and edible oil consumption, the prevalence of multimorbidity of appropriate consumption was significantly lower than that of excessive consumption or low consumption (*P* < 0.001). There also existed significantly differences in the prevalence among current-smokers (45.1%), ex-smokers (46.5%), and non-smokers (35.3%) (*P* < 0.001). For the consumption of fresh fruit, poultry meat, eggs and beans, and fish, the prevalence of multimorbidity increased with the decreasing frequency of consumption from group (often/always) to group (rarely/never) (all *P* < 0.05) (Table 2).

**Table 2:** Univariate factor analysis of multimorbidity

Variables	No Multimorbidity		Multimorbidity		χ^2^	*P*
	n	%	n	%		
Total	5282	78.8	1424	21.2		
Sex						
Male	2127	79.5	550	20.5	1.266	0.261
Female	3155	78.3	874	21.7		
Age (year)						
≤ 64	176	92.1	15	7.9	47.284	< 0.001*
65–69	3162	80.5	767	19.5		
70–74	1944	75.2	642	24.8		
Ethnicity						
Han	5134	78.8	1383	21.2	0.024	0.876
Non-Han	148	78.3	41	21.7		
BMI						
Underweight	91	89.2	11	10.8	64.783	< 0.001*
Normal	2141	40.5	427	30.0		
Overweight	2990	75.5	971	24.5		
Obese	60	80.0	15	20.0		
Marital status						
Married/Cohabitation	32	91.4	3	8.6	7.219	0.027*
Unmarried	4682	79.0	1241	21.0		
Divorced/ Separated	568	75.9	180	24.1		
Family history of hypertension						
No	4803	80.9	1137	19.1	136.24	< 0.001*
Yes	479	62.5	287	37.5		
Family history of diabetes						
No	4845	79.9	1219	20.1	66.016	< 0.001*
Yes	216	61.9	133	38.1		
Educational level						
Primary school or below	121	74.7	41	25.3	1.747	0.627
Junior middle school	678	78.7	183	21.3		
Senior middle school	1794	78.7	486	21.3		
Undergraduate or above	2689	79.0	714	21.0		
Occupation						
Agriculture	242	75.4	79	24.6	10.973	0.089
Industry	410	80.1	102	19.9		
Individual business and service industry	423	81.2	98	18.8		
Agency and business unit	483	81.6	109	18.4		
Retirement	2842	78.1	796	21.9		
Unemployment	533	80.3	131	19.7		
Other	349	76.2	109	23.8		
Annual income (¥)						
< 10,000	436	77.4	127	22.6	3.201	0.362
10,000 ~ 30,000	2197	78.0	620	22.0		
30,000 ~ 50,000	2200	79.6	565	20.4		
≥ ~ 50,000	449	80.0	112	20.0		
Physical exercise						
Every day	3895	79.3	1017	20.7	5.898	0.207
3-4 days/week	178	80.9	42	19.1		
2-3 days/week	158	79.0	42	21.0		
1-2 days/month	31	79.5	8	20.5		
Never	1020	76.4	315	23.6		
Sleep status						
Worse	54	71.1	22	28.9	19.187	0.001*
Poor	453	72.8	169	27.2		
Average	1583	78.6	432	21.4		
Good	2910	79.9	732	20.1		
Excellent	282	80.3	69	19.7		
Fatigueness status (time/week)						
<1	4210	79.4	1094	20.6	22.183	< 0.001*
1–2	838	79.0	223	21.0		
≥ 3	234	68.6	107	31.4		
Stay up late						
Often	166	77.6	48	22.4	4.675	0.197
Sometimes	391	81.6	88	18.4		
Rarely	995	80.0	248	20.0		
Never	3730	78.2	1040	21.8		
Smoking status						
Smoker	743	78.6	202	21.4	0.013	0.909
Non-smoker	4539	85.9	1222	85.8		
Status of alcohol drinking				0.0		
Drinker	654	80.2	161	19.8	1.215	0.27
Non-drinker	4628	78.6	1263	21.4		
Salt taste						
Salty	315	73.8	112	26.2	49.292	< 0.001*
Insipid	1028	72.9	383	27.1		
Appropriate	3939	80.9	929	19.1		
Edible oil taste						
Greasy	209	75.2	69	24.8	34.66	< 0.001*
Thin	974	73.2	356	26.8		
Appropriate	4099	80.4	999	19.6		
Carbonated drinks						
Yes	99	89.2	12	10.8	7.332	0.007*
No	5183	78.6	1412	21.4		
Fresh fruit consumption						
Often/Always	3852	80.6	925	19.4	75.884	< 0.001*
Sometimes	1055	78.0	297	22.0		
Rarely/Never	375	65.0	202	35.0		
Meat consumption (red meat)						
Often/Always	1470	80.4	359	19.6	5.625	0.06
Sometimes	2825	78.6	768	21.4		
Rarely/Never	987	76.9	297	23.1		
Meat consumption (poultry)						
Often/Always	1118	81.3	257	18.7	12.686	0.002*
Sometimes	3002	79.0	799	21.0		
Rarely/Never	1162	75.9	368	24.1		
Consumption of fish						
Often/Always	603	80.7	144	19.3	6.634	0.036*
Sometimes	3080	79.4	801	20.6		
Rarely/Never	1599	76.9	479	23.1		
Consumption of eggs and beans						
Often/Always	3068	80.0	769	20.0	8.208	0.017*
Sometimes	1780	77.5	518	22.5		
Rarely/Never	434	76.0	137	24.0		
Consumption of milk						
Often/Always	2353	79.1	621	20.9	0.412	0.814
Sometimes	1779	78.5	486	21.5		
Rarely/Never	1150	78.4	317	21.6		
Consumption of rice						
Often/Always	4270	79.2	1124	20.8	5.758	0.056
Sometimes	828	78.1	232	21.9		
Rarely/Never	184	73.0	68	27.0		

*P < 0.05

**Fig. 1:**
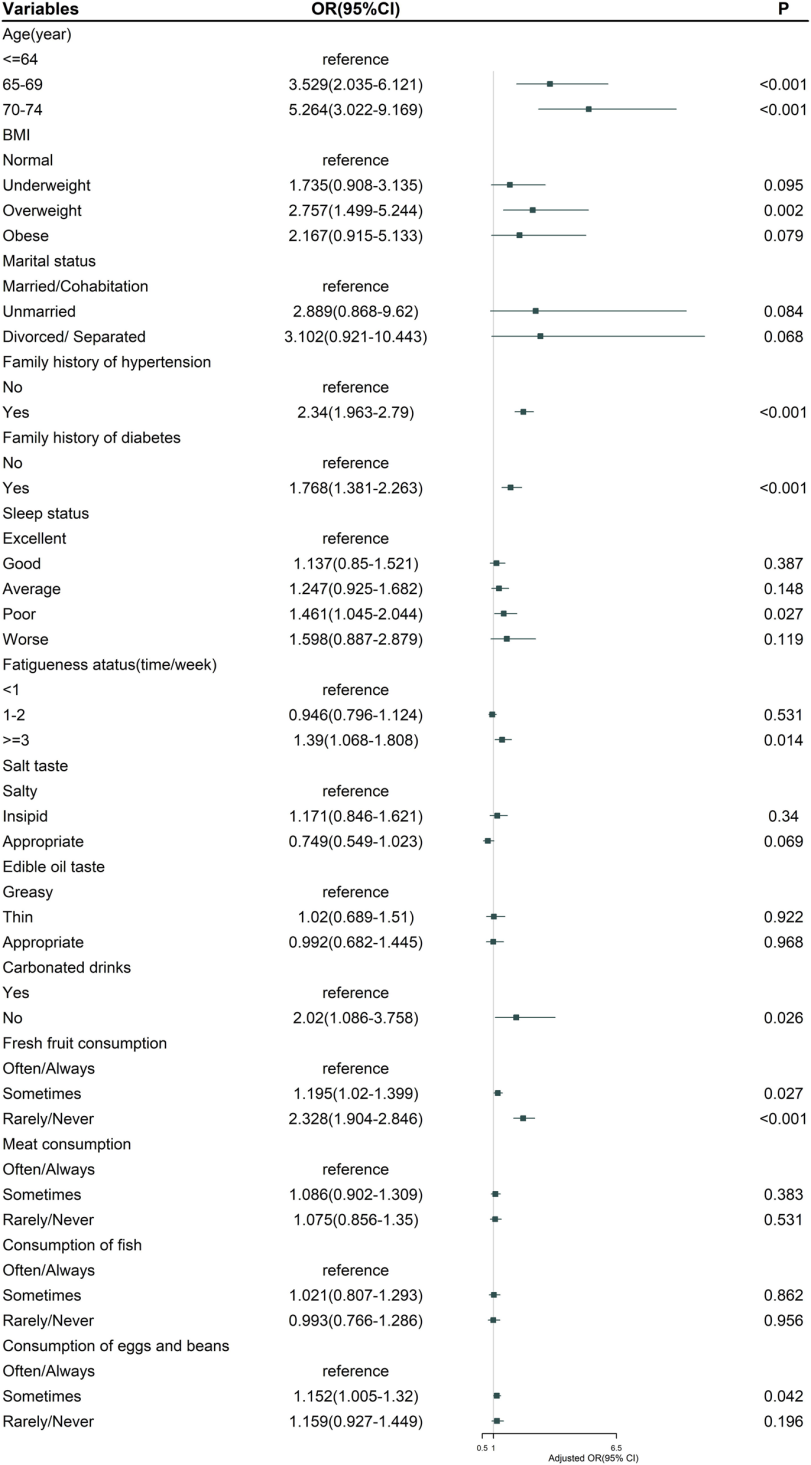
Multivariate logistic regression analysis of factors associated with multimorbidity

**Fig. 2:**
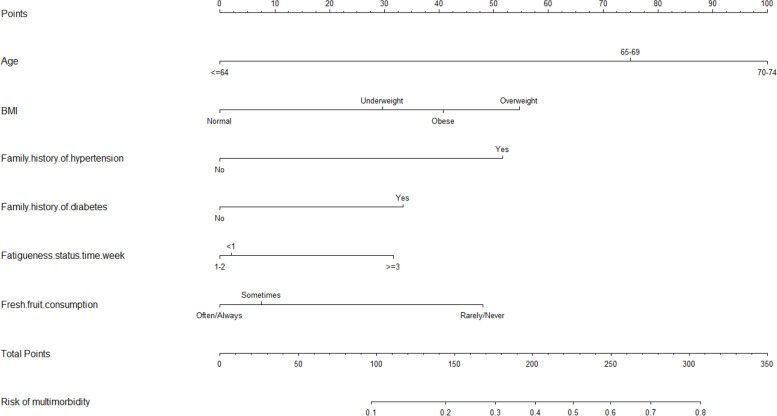
Nomogram for predicting multimorbidity risk. The nomogram was generated based on age, BMI, family history of hypertension, family history of diabetes, fatigue status, and fresh fruit consumption

We further used a multivariate logistic regression analysis, constructing a prediction model to validate multimorbidity-influencing factors. Data of the multiple logistic regression analysis, shown in Fig. 1, are visualized in the form of a nomogram to provide effective and reliable guides (Fig. 2). We identified that the increasing risks of multimorbidity were associated with independent factors (age, BMI, family history of hypertension, family history of diabetes, fatigue status, and fresh fruit consumption) (all *P* ≤ 0.01). Multimorbidity risks were related to aging (65–69 years old: OR = 3.53, 95%CI: 2.04–6.12; 70–74 years old: OR = 5.26, 95%CI: 3.02–9.17). Overweight participants had significantly high multimorbidity risks (OR = 2.76, 95%CI: 1.50–5.24). Family history of hypertension and family history of diabetes was significantly associated with high multimorbidity risks (family history of hypertension: OR = 2.34, 95%CI: 1.96–2.79; family history of diabetes: OR = 1.77, 95%CI: 1.38–2.26). Compared with the frequency of fatigue (< 1 time/week or 1–2 times/week), that (≥3 times/week) was associated with high multimorbidity risks (OR = 1.39, 95%CI: 1.07–1.81). For fresh fruit consumption, compared with participants eating fruits regularly, those eating rarely had higher risks of multimorbidity (OR = 2.33, 95%CI: 1.90–2.85). The C-index of the nomogram was 0.650.

## Discussions

In this paper, we documented that the prevalence of multimorbidity is 21.2% among the adult residents. In addition, the risks of multimorbidity are associated with age, BMI, family history of hypertension, family history of diabetes, fatigue status, and fresh fruit consumption.

The prevalence of multimorbidity in our study in 2019 is substantially lower than that in the study of Wang et al. in 2012 [34]. The decrease in prevalence of multimorbidity in northeastern China may be due to the implementation of chronic disease prevention and control strategies in decades. Actually, chronic disease prevention and control, supported by series projects focusing on chronic noncommunicable disease prevention and control, have been proceeding in northeastern China. With nationally spreading of 5G networks, healthcare systems conduct precise prevention and control for individuals with multimorbidity.

Aging has been widely considered to be associated with risks of multimorbidity [5, 35]. In agreement with other studies [36–38], our study also found that the prevalence of multimorbidity increased dramatically with aging. Moreover, consistent with other studies [25, 39, 40], our study found BMI influenced multimorbidity. Zhang et al. conducted a national investigation, finding that obesity is associated with the risk of multimorbidity in whole China [41]. Surprisingly, we corroborated that obesity was neither protect factor nor risk factor of multimorbidity in Northeastern China.

We identified the risk factors of multimorbidity (the family history of hypertension, family history of diabetes, and fatigue status [≥3 times/week]) in northeast China. These factors confer perception to connections implicated in multimorbidity. Thus, people with these three characteristics should pay more attention to their health and strengthen their awareness of prevention and control. In addition, for fresh fruit consumption, similar to the results of Ruel et al. [42], our results also showed that greater consumption of fruits appears to lower risks of multimorbidity.

Multimorbidity increases the risk of disability and mortality [43–45], necessitating the identification of influencing factors of multimorbidity. Moreover, our nomogram also provides effective and reliable guides for the risk-prediction, prevention, and control of multimorbidity. Overall, the adult residents with three characteristics (family history of hypertension, family history of diabetes, and fatigue status) are the population with high risk of multimorbidity. The three characteristics provide theoretical and precisely practical guidelines to prevent and control multimorbidity, such as controlling weight and increasing consumption of fruits.

There are strengths in this study, including the large sample size, comprehensive sociodemographic and behavioral characteristics, and region representativeness of northeast China. However, some limitations also exist. First, the causality between multimorbidity and risk factors could not be reflected in our cross-sectional design. Second, the data in this study were based on self-reported questionnaires; therefore, the accuracy of the reported results cannot be determined.

## Conclusion

In conclusion, the prevalence of multimorbidity is 21.2% among the adult residents of northeastern China. Sociodemographic indices (age, BMI, family history of hypertension, and family history of diabetes) and behavioral indices (fatigue status and fresh fruit consumption) increase the risks of multimorbidity. This study provides a necessary route to prevent and control multimorbidity in northeast China.

## Supplementary Information

**Additional file 1: Supplemental Figure 1.** Inclusion and exclusion criteria and selection process of participants.

**Additional file 2: Supplemental Table 1.** Definition of variables.

## Data Availability

All data generated or analysed during this study are included in this published article.

## Abbreviations

CI: Confidence Intervals
OR: Odds Ratios
