# Supplementary material for: Sociodemographic and behavioral influences on multimorbidity among adult residents of northeastern China
Source: BMC Public Health. 2022 Feb 18;22:342. doi: 10.1186/s12889-022-12722-y (PMC8855562; doi:10.1186/s12889-022-12722-y)
Supplement: Supplementary file 1 — Additional file 1: Supplemental Figure 1. Inclusion and exclusion criteria and selection process of participants. [file 12889_2022_12722_MOESM1_ESM.docx]

**Participants recruited in 2019**

**(n=8394)**

**Participants with complete information (n=7127)**

**Included participants in this study**

**(n=6706)**

**Exclude participants with incomplete information (n=1267)**

**Exclude participants with outliers (n=421)**

Supplemental Figure 1 Inclusion and exclusion criteria and selection process of participants.
