# Supplementary material for: Sociodemographic and behavioral influences on multimorbidity among adult residents of northeastern China
Source: BMC Public Health. 2022 Feb 18;22:342. doi: 10.1186/s12889-022-12722-y (PMC8855562; doi:10.1186/s12889-022-12722-y)
Supplement: Supplementary file 2 — Additional file 2: Supplemental Table 1. Definition of variables. [file 12889_2022_12722_MOESM2_ESM.docx]

**Supplemental Table 1. Definition of variables.**

| **Variables** | **Definition** |  |
| --- | --- | --- |
|  |  |  |
| Drinking | drinking alcohol at least three times a week for more than six months |  |
| Smoking | smoking at least one cigarette per day for more than six months |  |
| Physical exercise | physical exercise referred to one among flexible, aerobic, and anaerobic exercises. |  |
| Sleep status | sleep status was classified as worse, poor, average, good, or excellent, which an individual selected on the basis of intuition. |  |
| Fatigue status | fatigue status referred to weariness or exhaustion that an individual felt. |  |
| Stay up late | an individual who stay up late was one who go to bed after midnight. |  |
| Salt taste | salt taste was classified as salty, appropriate, or insipid. |  |
| Edible oil taste | edible oil taste was classified as greasy, appropriate, or thin. |  |
| Carbonated drinks | carbonated drinks referred to a combination of carbonated water and flavouring, sweetened by sugar or a non-nutritive sweetener. |  |
| Consumptions of fresh fruit | often/always (>3 days/week), sometimes (1-3 days/week), or rarely/never (<1 day/week). |  |
| Consumptions of meat (red meat and poultry) | often/always (>3 days/week), sometimes (1-3 days/week), or rarely/never (<1 day/week). |  |
| Consumptions of fish | often/always (>3 days/week), sometimes (1-3 days/week), or rarely/never (<1 day/week). |  |
| Consumptions of eggs and beans | often/always (>3 days/week), sometimes (1-3 days/week), or rarely/never (<1 day/week). |  |
